# Applying Systems Thinking to Inform Decentralized Clinical Trial Planning and Deployment

**DOI:** 10.1007/s43441-023-00540-2

**Published:** 2023-06-30

**Authors:** Lidia Betcheva, Jennifer Y. Kim, Feryal Erhun, Nektarios Oraiopoulos, Kenneth Getz

**Affiliations:** 1 Judge Business School, University of Cambridge, Cambridge, CB2 1AG UK; 2 Tufts Center for the Study of Drug Development, Tufts University, Boston, MA 02111 USA

**Keywords:** Decentralized clinical trials (DCTs), Systems thinking, Stakeholder analysis, Pain points, Adoption and implementation of DCTs

## Abstract

Recently, there has been a growing interest in understanding how decentralized clinical trial (DCT) solutions can mitigate existing challenges in clinical development, particularly participant burden and access, and the collection, management, and quality of clinical data. This paper examines DCT deployments, emphasizing how they are integrated and how they may impact clinical trial oversight, management, and execution. We propose a conceptual framework that employs systems thinking to evaluate the impact on key stakeholders through a reiterative assessment of pain points. We conclude that decentralized solutions should be customized to meet patient needs and preferences and the unique requirements of each clinical trial. We discuss how DCT elements introduce new demands and pressures within the existing system and reflect on enablers that can overcome DCT implementation challenges. As stakeholders look for ways to make clinical research more relevant and accessible to a larger and more diverse patient population, further robust and granular research is needed to quantify the impact of DCTs empirically.

## Introduction

There are many hurdles to overcome when identifying, enrolling, and retaining study participants in clinical research. These hurdles are associated with numerous factors, including patient access and willingness to participate; protocol demands and eligibility constraints; and physician willingness to refer and facilitate participation. Although 85% of people are willing to participate in clinical trials, for example, only a fraction do [1]. It has been estimated that less than 10% of eligible adult cancer patients participate in clinical trials [2]. In a typical phase III clinical trial, more than one-third (37%) of clinical research sites under-enroll, and 11% fail to enroll even a single participant [3]. Moreover, due to dropout rates—on average estimated as high as 30% [4]—participant retention can compromise study results and carry significant financial consequences. In fact, the average cost per patient has risen by 70% in the past 3 years [5]. Further, recruitment and retention problems can delay clinical trial completion, costing sponsors up to $8 million daily [6] in lost drug sales.

In conventional clinical trials, participants visit investigational sites, often located in large medical facilities in metropolitan areas. The centralization of operations in such locations far away from where potential participants live may hinder participation [7]. To illustrate, 70% of potential participants in the U.S. live more than 2 h away from the nearest study center [8]. A 2019 study assessing patient engagement in clinical trials with more than 12,000 respondents identified travel to and from sites as the top study participation burden, with 29% indicating that it was “somewhat” or “very burdensome” [9]. In addition, these geographical constraints disproportionately affect underprivileged groups (e.g., people from lower socioeconomic groups may not be able to take time off work or afford to travel long distances for trial visits), particularly those with intersecting identities (e.g., racial minority women) [10]. This barrier may contribute to the lack of representation in clinical trials, jeopardizing external validity and generalizability of results and ultimately resulting in ineffective or even harmful drugs among certain demographic groups [11].

Clinical trial complexity has also grown significantly during the past decade, placing a substantial burden on investigative sites. Since 2010, for example, the average number of endpoints in phase II and III protocols has increased 27%, and the average number of procedures performed per patient visit has increased 22% [12, 13]. However, this complexity has run counter to patient expectations of greater convenience of care [14]. Moreover, growing interest in real-world evidence (RWE) in clinical trial data generation has called for data collection from the point of routine care in addition to locations outside the brick-and-mortar boundaries of the healthcare system [7].

The COVID-19 pandemic exposed the vulnerabilities of the conventional, site-centric clinical trial design in several ways [15]. First, the redirection of healthcare resources and staff to care for COVID-19 patients led to staff shortages, which were exacerbated by staff falling ill with COVID-19. Second, particularly early in the pandemic, on-site visits by clinical research associates were limited by the shift to a virtual setting due to government restrictions and regulatory guidance [16]. Access to sites was affected by geographical differences and the state of the pandemic. One survey of organizations within the sector showed that between 35 and 80% of sites were inaccessible [17]. Third, travel restrictions and stay-at-home orders prevented participants from visiting sites for regular dosing and assessment. Some contracted COVID-19, while others skipped visits out of fear. According to a poll, in May 2020, nearly half of Americans (48%) said they missed or delayed receiving medical care due to the pandemic [18], which was a key concern as missed visits and “out-of-window” visits led to protocol deviations that jeopardized data integrity. Finally, the pandemic also created shortages of ancillary supplies for clinical trials due to disruptions in the supply chain and logistical challenges involving transportation caused by lockdowns [19]. These issues affected subject enrollment, protocol adherence, trial operations, and data collection [20]. One analysis found an 80% year-on-year decrease in new patient enrollment for April 2020 [21]. Pharmaceutical decision-makers triaged trials by devoting resources to the most promising studies and those with the least risk for patients [17]. This decision ultimately led to the postponement or cancellation of planned studies and, in some cases, suspension or termination of ongoing studies [21].

To keep clinical trials going, minimize the risk of transmission of COVID-19, and preserve the continuity of care, data collection, and data integrity, many sponsors quickly deployed remote and virtual approaches (i.e., decentralized clinical trial (DCT) solutions), including eConsent, remote patient monitoring, data collection via wearable and mobile devices, and at-home assessments [22]. Consequently, DCT deployments soared during the COVID-19 pandemic. The number of clinical trials with virtual and/or decentralized elements surpassed 1000 in 2021 (a 50% increase compared with 2020), and 1300 trials were forecasted to initiate in 2022 [23].

DCT use in clinical trials promises to address a number of key drug development challenges. In addition to improving patient access and participation convenience, DCT solutions may also improve patient adherence to the protocol and may increase overall retention rates [24]. DCTs enable clinical research data to be collected more easily and faster, offering the opportunity to interrogate and draw insights from the data sooner, reduce the number of patients required, and increase statistical power [8]. The deployment of remote and virtual solutions may also offer operational efficiencies through the automation of select manual data collection tasks, more frequent communication and interaction with study volunteers, and more productive investigative site personnel [25–27].

Anecdotal reports and early case examples suggest that the promise of DCT use in clinical trials is being realized. Sponsors, contract research organizations (CROs), and DCT vendors have reported positive results with DCT deployments [28–31]. For example, Sarraju et al. [32] implemented a virtual study among atrial fibrillation patients, consisting of virtual recruitment via social media and virtual monitoring using a mobile application and sensors. Results showed high adherence, positive study engagement outcomes, and willingness to continue in a larger trial. Hilderbrand et al. [27] conducted a 1000-patient virtual clinical trial in just seven months at a fraction of the cost of traditional site-based recruitment, demonstrating the benefits associated with reducing recruitment cycle times, and overall improvement in patient experience as patients reported satisfaction and willingness to move forward with the study. Overall, these cases exemplify the feasibility and benefits of a decentralized approach.

With growing deployment experience, some sponsors and CROs have reported challenges introduced by DCTs, including increasing clinical trial execution complexity, longer study start-up durations, and higher costs associated with installing technologies and infrastructure, offering training to site personnel and study volunteers, and providing technical support [33].

As more is learned—both positive and negative—about DCT use in clinical trials, sponsors and their collaborative partners face great difficulty in weighing benefits and risks and anticipating operating challenges. In this paper, we apply systems thinking to guide sponsors and CROs in comprehensively considering remote and virtual solutions—their advantages, pain points^1^ addressed and introduced, and trade-offs—in protocol design and execution planning processes.

## Methods

### Defining DCTs

DCTs are broadly defined as clinical trials wherein recruitment and data collection are not restricted to centralized location(s) as is typical for conventional trials. Table 1 summarizes the more common DCT solutions in use today.

**Table 1:** DCT Solutions

Element	Description
Digital health technologies	Technologies that track, monitor and capture participant health data and provide healthcare services including mobile device apps, wearables, bring your own device (BYOD), etc.
eConsent	Process that provides information about a study and obtains informed consent from study participants through a digital format
ePRO/eCOA	The capture of clinical outcome assessments (COA) such as participant reported outcomes (PRO) data through the use of electronic devices (e.g., e-diaries)
Virtual visits/eVisits/teleconsults	Consultations by remote telecommunications between a site investigator and a participant that take the place of in-person site visits
Mobile clinics and home health	Interventions and data collection by home healthcare professionals (HCPs) conducted in clinical trial visits that take place in a participant’s home, workplace or mobile clinic in their community
Direct-to-patient IMP shipping	Delivery of IMP from a site, depot or pharmacy directly to a participant’s home as well as the collection of specimens for laboratory testing and unused IMP for reconciliation and destruction

Among DCT deployments, there are two main variations: (1) DCTs that are entirely remote (full DCTs); and (2) DCTs that are partially remote (hybrid DCTs). Full and hybrid DCTs are achieved using telemedicine, digital health technologies, and approaches centralized around patient accessibility and convenience. The degree of decentralization can be assessed on two dimensions: the *locality* of the data capture (ranging from on-site research facilities to remote locations) and the *methods* for data collection (ranging through the use of intermediaries to fully virtual) [7].

### Analysis that Applies Systems Thinking

We applied systems thinking^2^ to assess DCT deployment and its comprehensive interaction with the larger and complex process of clinical trial execution [36]. Systems thinking takes a holistic perspective when considering problems and their solutions [37].

A recent and relevant application of this approach is the “Engineering Better Care” systems framework that considered four interrelated perspectives (people, systems, design, and risk) to evaluate health and care design and improvement initiatives in an iterative and holistic way [38]. Importantly, the framework considers stakeholders and their needs, the system architecture, a range of possible solutions that would help meet the needs of the system, and an assessment of what could go wrong/and can be improved. Applying a similar “whole system” approach, we consider how decentralization impacts clinical development.

**Figure 1:**
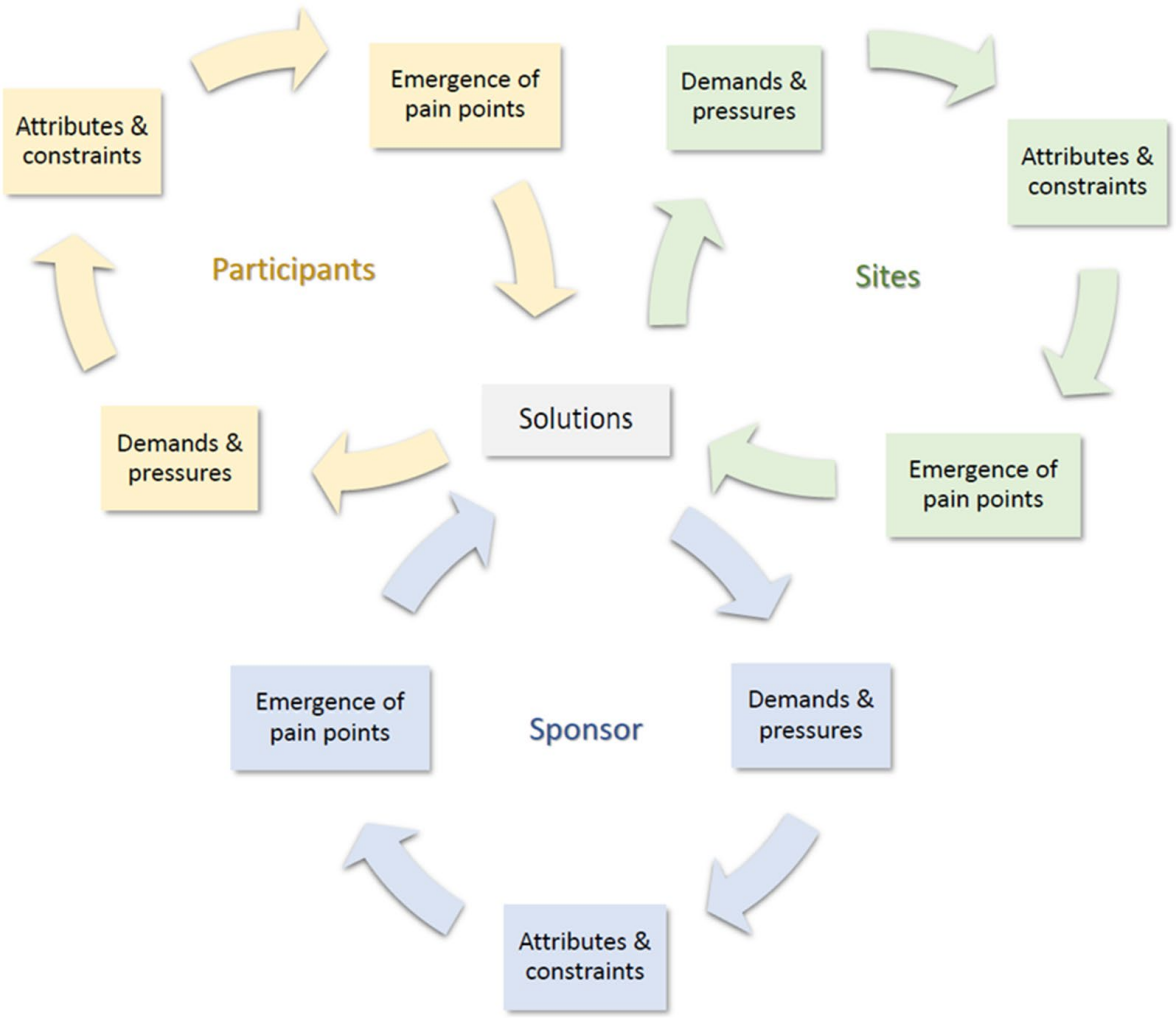
A Systems View of Pain Points for Participants, Sites, and Sponsors

Since a system is defined by its interconnections, a change in one element of the system invariably impacts other area(s) of the system. Thus, when a solution is introduced to alleviate a pain point for one or more stakeholders, it may introduce *new* system demands and pressures for other stakeholders. The emergence and alleviation of stakeholder pain points result in an iterative process that introduces new solutions, which then may add *new* system demands and pressures that lead to different pain points. Figure 1 captures this iterative process for DCT deployments (and its impact on participants, sites, and sponsors). It is worth mentioning that the system may include a wide range of stakeholders, such as CROs, investigative sites, home HCPs, local care providers, regulators, institutional review boards, patient advocacy groups, payers, technology providers, couriers, and support services. The choice of focal stakeholders in the system depends on the purpose of the analysis and the goals one is striving to achieve.

This analysis helps to identify the appropriateness of DCT use in different settings based on the system's characteristics, such as the characteristics of the patient population, the disease, and the capacity and infrastructure availability. Various factors make certain studies or indications prime candidates for incorporating DCT solutions, which we will discuss in more detail.

## Results

**Figure 2:**
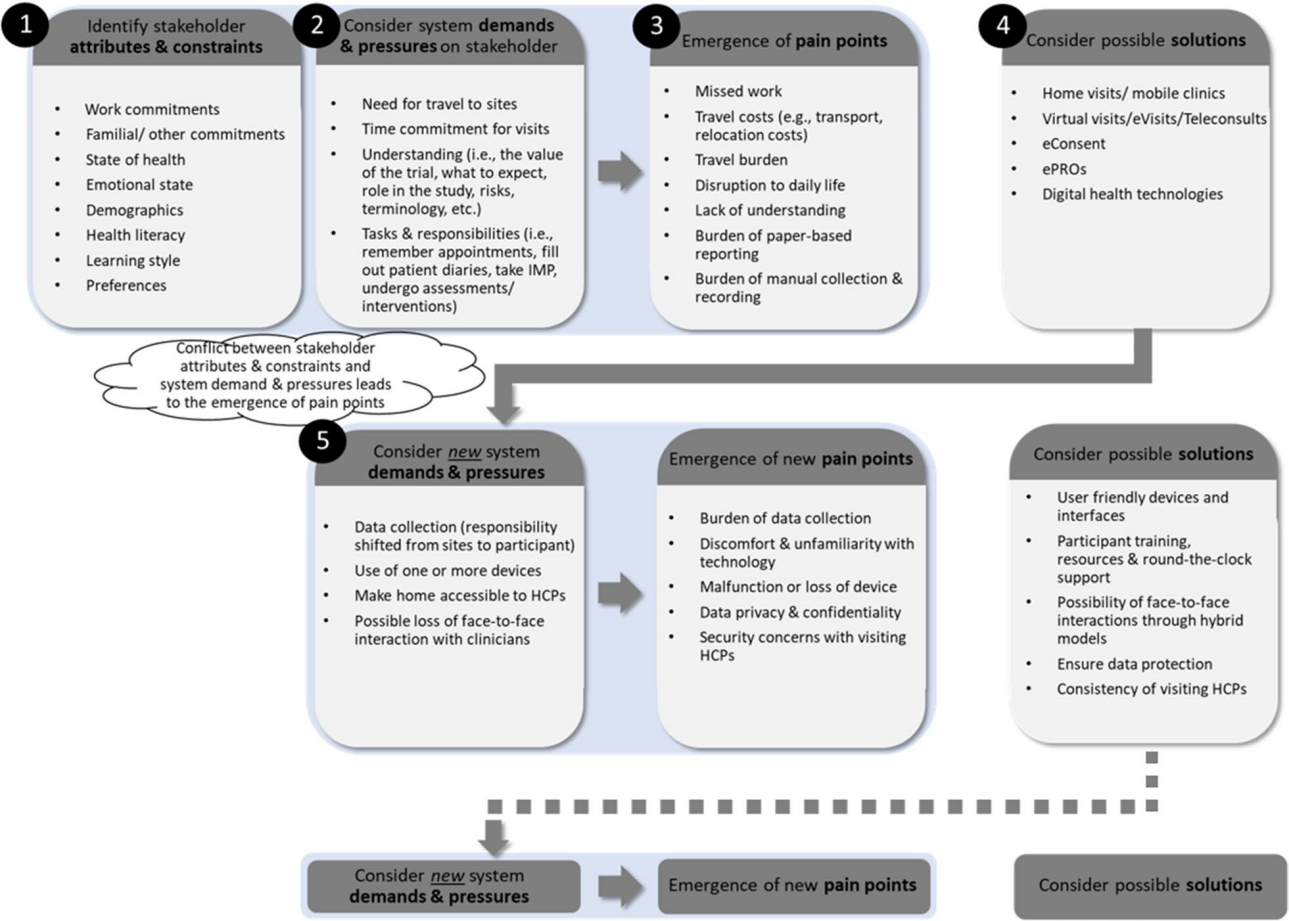
The Process of Emergence and Alleviation of Pain Points for Clinical Trial Participants

Figure 2 presents the results of our assessment on the impact of DCT solutions using a systems thinking approach applied to a single stakeholder, the study participant. Based on literature review and industry reports, we aim to cover many first-order effects.^3^

Table 2 supplements the analysis by providing key advantages, disadvantages, and considerations for DCT elements that may serve as solutions to stakeholder pain points in this reiterative process.

**Table 2:** DCT Solutions—Advantages and Challenges Mapping

Element	Potential Advantages	Potential Challenges and Considerations
Digital health technologies	- **Improved patient outcomes**^a^ - Generation of more comprehensive **real-time data** in a **real-world** setting - Rapid handling of issues such as adverse events through **real-time monitoring** - **Reduced need for on-site visits** (this has time and effort implications for participants and site personnel) - **Enhanced patient experience**^b^ - **Increased participant convenience**: e.g., with wearables, participants do not have to manually collect and record data - **Higher patient engagement**: e.g., patients can be more active in their care and better understand how their behaviors can affect their health in real-time [52]	- Management, interpretation and analysis of **large amounts of data** - **Incorrect use/malfunction/loss** of device - **Increased participant burden**: shifting burden of data collection from site personnel to participant (especially worrisome for naive trial participants or if participant needs to use several devices for a study) - Level of **oversight and environmental control** - Ensure **data privacy and security** - **Cost** considerations: e.g., provisioning devices requires purchase and shipping, storage, distribution (to sites and participants), maintenance and replacement costs for the sponsor [53] - **Regulatory** considerations: technology needs to be qualified to capture high-quality clinical data - **Operational** considerations: e.g., how to incorporate technologies into the protocol, appropriateness of technologies for the study, technology obsolescence (especially for longer studies), etc.
eConsent	- **Improved traceability** through date and time stamps - **Improved participant comprehension**^c^ - Consent in the **comfort** of participants’ homes^d^ - **Reduced errors** such as incomplete consent forms leading to protocol deviations - **Less administrative burden** and paperwork for sites - **Ease of updating** consent forms and obtaining re-consent after protocol amendments	- **Resistance to adoption** by healthcare professionals - **Financial and time investment** (such as site personnel training) - **Changes in existing processes and workflows** - **Evaluation of vendors**’ solutions and support (provided to participants and sites)
ePRO/eCOA	- Eliminates or **reduces missing/inappropriate/out-of-range responses** through limits and controls on data entry - **Ensures timely completion** by reducing “parking-lot compliance” and forward filling through alerts to participants and specified time windows for data entry^e^ - **Reduces patient and site burden** associated with maintaining paper-based diaries - Permits **faster data processing and analysis** through automatic upload of data. This can also quickly alert sites when there is a reduction in compliance based on ePROs	- **Technical problems** potentially leading to the loss of data, reduction in participant compliance and/or participant experience - **Time, cost, and resources** required for **training and supporting participants** in using electronic diaries - Differences in **participants’ comfort with digital technologies** and computer literacy
Virtual visits/eVisits/teleconsults	- **Reduced need for participant travel** (this has implications for **reducing participant burden** as well as **enhancing participant reach**, i.e., for those in certain geographic areas, those with disabilities, etc.) - **Improved infection control** (especially relevant for studies involving immunocompromised participants) - Potential for **better assessment**^f^	Ftouni et al. [54] provide a systematic review of the challenges with telemedicine. These include **technical issues** (including **lack of universal access** to technology, poor connection which hampers communication), **privacy and confidentiality** (e.g., cybersecurity risk), **lack of physical examination** (especially for complex procedures and specialized assessments), special populations (e.g., **digital literacy** in certain age groups, **demographic disparities**), **training** of providers and patients, **need for face-to-face interaction** in establishing rapport (especially when subjects are participating in a clinical trial for the first time)
Mobile clinics and home health	- Enhanced **patient centricity**: less disruption to participants’ lives - **Reduced burden** (of performing more routine activities such as blood draws) on sites - **Increased access** to clinical trials to underserved communities - Home administration of investigational medicinal product (IMP) that is **representative of real-world administration** once approved	- **Feasibility** of home care (dependent on phase, type of indication, need for equipment and physical infrastructure, patient population) - Home HCP’s **qualifications, scope of practice and training**: e.g., Good Clinical Practice, adverse event reporting, data protection - **Patient preference**: e.g., security concerns regarding visiting home HCPs - **Operational** considerations: e.g., sample stability in transit, technology or equipment failure (mobile or home HCPs do not have access to immediate expert assistance/supplies), responsibility for oversight of home HCP - **Consistency** of data collection across sites and home HCPs - Sponsor’s **cost** considerations (dependent on type of HCP needed, training, etc.)
Direct-to- patient IMP shipping	- **Facilitates the conduct of trial visits outside of sites** (i.e., through home visits, virtual visits, etc.) which has implications for participants’ need to travel, disruption to lives, patient experience, and participant recruitment and retention	- Participant **confidentiality and privacy** - **Feasibility**: depends on IMP’s stability and shelf-life, risk profile, dosing frequency, route of administration, special preparation vs. ready-to-use, ease of administration - **Accountability/traceability/chain of custody** - Ensure IMP is delivered in **good quality** (drug integrity and temperature control) - **Participant compliance**: e.g., if IMP is self-administered, is patient storing/administering/safeguarding/disposing the drug correctly? - Compliance with regional, state, national **laws and regulations** (where IMP is dispensed and received) - **Costs**: e.g., shipping, inventory, wastage, specialized couriers, ability to pool supply - **Coordination:** e.g., arrival window of delivery may need to be coordinated with home HCP visit to administer the IMP

^a^As summarized in [55], various studies have shown such technologies are associated with enhanced outcomes such as greater weight loss (in a weight loss intervention study) and improved drug adherence

^b^In comparing BYOD to paper records and a provisioned device, Byrom et al. [56] find 94% of subjects would definitely or probably be willing to download an app onto their own mobile device for a forthcoming clinical trial with 45% expressing that BYOD would be more convenient compared with 15% preferring a provisioned device. Note that BYOD eliminates the need to carry and maintain a second device for the duration of the study

^c^Through the use of multimedia such as images, video, audio (e.g., in multiple languages), quizzes, electronic glossaries, etc. the consent process can be interactive, engaging and tailored to the trial subjects’ demographics and participants’ learning styles [57, 58]

^d^This can allow participants more time for consideration, thereby reducing the pressure for immediate consent and allows for family and caregivers to be involved in the review process [59]

^e^ “Parking-lot compliance” refers to the notion of participants filling in their entire diary immediately prior to a study visit whereas forward filling refers to participants entering the data prior to the scheduled time [60]

^f^Telemedicine can allow specialists to see participants in their home environments, e.g., allergists may be able to identify clues in the patients surroundings that cause allergies [61]

*Steps 1–3*: *The emergence of pain points.* Clinical trial participants have individual *attributes* such as their state of health, demographics, and personal preferences, as well as *constraints* such as work, familial, and other commitments. Participation in a traditional site-centric clinical trial requires them to travel to sites for visits and develop an understanding of the trial (i.e., their role in the trial and the associated risks and relevant terminology). Moreover, participants have roles and responsibilities such as remembering and attending visits, undergoing assessments, and filling out patient diaries, to name a few. Resulting from the system design/architecture, these system *demands and pressures* may conflict with participants’ attributes and constraints. For instance, consider a working single parent who may need to travel long distances to reach a site. Travel demands conflict with the participant’s work and familial commitments, leading to a pain point. Furthermore, this pain point can be particularly pronounced for participants from certain demographics (e.g., low socioeconomic status and those living in rural areas).
*Step 4*: *Possible solutions to alleviate pain points.* There are many alternative solutions to alleviate stakeholder pain points. For example, financial compensation can be provided for missed work, reimbursements and stipends can be offered for travel costs incurred, and special travel can be arranged for participants with mobility issues. However, many of the pain points specified in Fig. 2 can be alleviated through DCT elements (i.e., home visits and mobile clinics, virtual visits, eConsent, ePROs, and digital health technologies).
*Step 5*: *Introduction of new system demands and pressures.* Many DCT solutions—including the use of mobile devices and home-based assessments—transfer execution responsibility away from what was historically handled by site personnel to the participants themselves. If this demand conflicts with participants’ attributes (i.e., digital literacy, demographics) and/or preferences, this may lead to the emergence of pain points. In turn, new solutions may need to be introduced to address emerging pain points (e.g., discomfort with technology can be alleviated through participant training, round-the-clock support, etc.). Successive solutions present new demands and pressures, leading to new pain points.

*Iterative steps: Minimization and management of pain points.* By repeating Steps 2–5 multiple times, decision-makers can evaluate the emergence and alleviation of pain points in applying various solutions.

The resulting analysis and insights can allow decision-makers to identify ways to minimize or mitigate stakeholder pain points so that, ultimately, more value can be created from implementing DCT solutions.

DCT solutions identified for the participant analysis in Fig. 2 also address and alleviate pain points faced by sites (e.g., administrative burden and paperwork, errors from manual data entry, the workload associated with menial tasks stemming from site visits, etc.) and sponsors (large investigator grant payments, recruitment and retention issues, among others). However, by the systems view approach, such solutions may also introduce new demands and pressures onto stakeholders. Demands and pressures should again be assessed alongside all stakeholders’ attributes and constraints, and a similar process to the one illustrated in Fig. 2 should be performed for all key stakeholders.

Table 3,^4^ provides a sample of the new demands and pressures experienced by participants, investigative sites, and sponsors from the introduction of various DCT solutions. DCT deployments require the use of new technologies, new stakeholders and new stakeholder roles in the provision of care, and data collection outside of traditional investigative sites. Thus, demands and pressures arise from the two dimensions of decentralization (digitalization and locality) [7] introduced earlier, and can be broadly grouped into four categories: the introduction of new technology, reliance on staff outside of sites, a greater reliance on the participants themselves, and changes to the supply chain.

**Table 3:** A Sample of the New Demands and Pressures Introduced by DCT Solutions and Resulting Pain Points Faced by Sponsors, Sites, and Participants

Demands and Pressures from DCT Solutions	Stakeholder	Pain Point
Introduction of new technology	Sponsor	- Payments to technology vendors - Expensive technology - No available technology to meet needs - Implementation costs - Training and upskilling needs of sites, CROs, home HCPs, other personnel - Data integration (of data coming in from multiple sources) - Inconsistent state telemedicine laws - Ensuring privacy and confidentiality
	Sites	- Training burden - New or altered standard operating procedures - Additional workload (esp. with hybrid trials) - Oversight: Verifying participants’ identities - Oversight: Adequately gauging participants’ understanding - Ensuring privacy and confidentiality
	Participant	- Difficult and/or inconvenient to use - Malfunction or loss of device - Data privacy and confidentiality
Reliance on mobile clinic and home HCP vendors	Sponsor	- Payments to mobile clinic/ home HCP vendors - High turnover of home nursing staff - Varying medical qualifications of mobile/home HCPs - Inconsistencies in knowledge of the protocol of mobile/ home HCPs
	Sites	- Oversight of source documents - Acceptance of external staff (e.g., home HCPs) - Worry that they will be cut out of the process (affecting investigator payments)
	Participant	- Security concerns and discomfort with home visits
Reliance on participants	Sponsor	- Oversight: Adverse event reporting through remote technologies - Oversight: Ensuring data integrity and safety monitoring
	Sites	- Oversight: Ensure digital health technologies used by participant and not someone else - Oversight: Ensure digital health technologies used correctly and as intended - Oversight: Ensure data is recorded properly and truthfully
	Participant	- Desire for face-to-face interactions with HCPs and clinical experts - Burden of data collection - Concern of being unequipped for new responsibilities and tasks
New supply chain vendors	Sponsor	- Payments for direct-to-patient IMP shipping/couriers - State differences for direct shipping of IMP to participants

Multiple detailed and holistic iterations designed to alleviate stakeholder pain points culminate insight into primary advantages such as a reduction in site burden, enhanced access and increased diversity, improved external validity of findings, and possible cost savings. However, the demands and pressures imposed by DCT solutions on clinical trial systems also amount to several overarching challenges, including potential inequalities, privacy and data protection issues, and complex operational requirements.^5^ Despite these challenges, enablers (such as growing regulatory agency commitment and digital advances) moderate the degree to which the new demands and pressures actualize into pain points and, therefore, continue to spur demand for DCT solutions. A detailed discussion on the advantages, disadvantages, obstacles, and enablers associated with DCTs can be found in the Appendix.

## Discussion

What becomes apparent through systems thinking analysis is that the appropriateness of DCT use differs depending on system characteristics. Various factors make certain studies or indications prime candidates for incorporating DCT solutions. Discomfort and unfamiliarity with technology is a participant pain point associated with the digitalization component of DCTs. For sites and sponsors, important considerations include constraints relating to the specifics of the study (e.g., the therapeutic area, the phase of the trial, the incidence and prevalence of disease, etc.), national and international regulatory environment, existing resources and infrastructure (e.g., staff, equipment, technology, competencies, procedures, etc.), and the budgets for clinical trial conduct.

Clinical Trials Arena has tracked the distribution of different DCT categories by therapy area [40]. The analysis finds that telemedicine and remote monitoring are the most widely accepted DCT components across therapy areas. Telemedicine has been used the most in infectious disease and oncology trials, while, perhaps not surprisingly, remote monitoring (using sensors, device, and trackers) has been prominent in cardiovascular, central nervous system, and metabolic disorder trials. Moreover, due to the regulatory and operational requirements brought about by the COVID-19 pandemic, COVID-19 drug trials were the most likely to use remote drug delivery and remote nursing [41]. The research finds that dermatology and women’s health trials have most often incorporated ePROs, eCOAs, or eConsent. It is noted that the complexity of disease may limit the uptake of such components in oncology trials. Furthermore, data from eCOA may be less important for certain therapy areas, such as cardiovascular and metabolic disorders, that are more concerned with physiological measurements as key endpoints rather than reported outcomes [40]. This highlights a key point alluding to the quote “just because you can, doesn’t mean you should*.*” Even though a DCT element may be easily incorporated into a trial, the appropriateness to do so depends on the value it creates for the system.

Demands and pressures imposed by DCT solutions may be more aligned with certain attributes, thus leading to fewer (and less pronounced) pain points. For example, the therapeutic area and the types of assessments required for the trial are two critical constraints. Degenerative conditions whereby travel for even short distances is especially burdensome [42] or areas such as stroke management, where patients can manage their disease condition relatively easily, and dermatology, in which telemedicine (and video consultation) is suitable and already well-utilized [39], may be most appropriate for DCT solutions. Similarly, sleep studies conducted at home can provide more informative data and better facilitate patient preferences [43]. Such studies may be good candidates for the early adoption of most DCT elements. They can also pave the way for other indications by exemplifying the implementation processes and lessons learned.

Clinical trials in oncology and infectious diseases, on the other hand, in which the safety of the investigational drug is not well characterized and require tests that can only be performed in medical facilities (e.g., magnetic resonance imaging) [44] are unfavorable candidates for *complete*^6^ decentralization. In these cases, DCTs should be treated as one of the many resources that drug development stakeholders can add to their toolbox, and the decisions in choosing the right DCT elements for a hybrid trial approach become paramount.

Sites and sponsors will also consider the operational requirements of the study (e.g., dosing frequency, method of administration, investigational drug storage requirements, to name a few) as well as whether there exists appropriate and validated technology and if the infrastructure is (or can easily be) established. Moreover, the regulatory environments in which DCTs will take place must be carefully evaluated. Different geographies may have different laws and regulations regarding telemedicine and direct-to-patient shipping of IMP and be more or less receptive to trial decentralization.

In thinking about how to scale and ensure the longevity of DCTs, it is crucial that appropriate solutions are deliberately selected and tailored to align with the specifics of the system prior to implementation. This contrasts the early phases of DCT use in the pandemic where, to a certain extent, solutions were “shoehorned” into existing systems [45]. A key aspect of this is a consideration of the partner ecosystem. Looking across the system, tensions may appear when the costs and benefits stemming from DCT implementation are not shared equally. One question that arises is whether the industry is stretched in two directions: offering patient choice versus the pursuit of operational excellence (i.e., executing trials faster and cheaper) [46]. The greater the alignment between such conflicting factors, the easier it is to ensure the sustainability of DCTs. This may necessitate building certain capabilities causing stakeholder roles to shift (e.g., consider, for example, the rising industry demand for data scientists) [45].

It is important to recognize that to minimize implementation challenges, trade-offs and pain points need to be recognized and mitigated. The systems approach presented in the paper offers stakeholders a way of holistically examining the impact of DCTs on the system: the implications for themselves and as well as on their partners. Each stakeholder needs to ask themselves a series of questions: (i) What problem do we want to solve?; e.g., increase participation in a clinical trial, reduce costs, speed up data collection, etc. (ii) Who are the stakeholders and partners? Which organizations could we approach? (iii) Why do we approach this problem using a DCT?; e.g., disease profile, patient profile, etc. (iv) How can we overcome the challenges? What are we good at, and what do we need to improve?

## Conclusion

Although the many possibilities offered by DCTs underscore strong industry interest in trial decentralization, without detracting from the promise of DCTs, we urge caution in the widespread application of DCT solutions absent a thorough systems-oriented consideration of their impact on the clinical trial operating environment.

The current operating environments calls for heightened awareness and demand for solutions that can simplify the clinical development process for staff and patients. There is no doubt that DCTs will be a part of that effort. A recent survey found that most biopharma respondents viewed DCTs favorably [47]. However, how DCTs are implemented and incorporated into existing clinical research paradigms remains to be seen. A*t the current state* of DCT adoption, many organizations, surveys, and roundtables reveal that customized hybrid trials are thought to be the most viable option [27, 32]. We believe a “one-size-fits-all” approach is inappropriate even as DCT adoption reaches maturity. As seen through the systems thinking framework, any such incorporation has wide-ranging impacts on key stakeholders and should be carefully considered. DCT solutions should be treated as one of the many tools that drug development stakeholders can add to their arsenal as they look for ways to make clinical research more relevant and accessible to a larger and more diverse^7^ patient population.

To ensure that decentralization can enable diversity in clinical research, DCT solutions (and the resulting demands they place onto a diverse participant population) should be assessed to confirm that patients from diverse backgrounds are being included. Moreover, digital elements should be coupled with high-quality support and training to increase participants’ comfort and willingness to use technology. Importantly, clinical trials need to be designed to include accommodating options that meet a variety of patient preferences (and factor in various considerations such as participants’ socio-economic status). The resulting downstream impact (e.g., effect on data collection and data quality) of this level of customization should also be evaluated. Reinforced by a recent push from regulators [50, 51] to increase diversity in clinical testing, well-designed studies incorporating DCT solutions may offer a way to address under-representation in clinical research by removing some of the barriers associated with traditional clinical trials [10].

Systems thinking can assist decision-makers in assessing the system effects of DCTs. In choosing the best candidates and elements for DCT implementation, the framework may reveal different insights for different systems corresponding to their unique characteristics and, importantly, can be used to shed light on the appropriateness of DCT use and where there may be shortfalls. If a DCT solution leads to the emergence of more non-actionable pain points than the number of pain points it alleviates, one should be cautious in its steadfast acceptance and implementation. This approach can help the industry adopt solutions with higher chances of success and create best practices for implementation early on. This will pave the way for introducing potentially more complex elements and solutions by solving any technology and infrastructure-related issues upfront.

Recent trends indicate growing regulators’ commitment toward DCTs, laying the groundwork for regulatory guidance and oversight on the adoption of DCT [22]. Indeed, the COVID-19 pandemic has resulted in increased and quickly evolving regulatory acceptance of decentralized interventions. Furthermore, the pandemic has helped improve attitudes toward digital health solutions and has heightened stakeholder comfort levels with digital technologies, which can undoubtedly reinforce the continued adoption of DCTs where appropriate.

In conclusion, despite the enthusiasm surrounding the adoption of DCTs in clinical research, more robust research is needed to quantify the impact of DCTs empirically. The systems thinking framework provides a systematic and reiterative way to identify pain points and assess possible solutions through DCT implementation. A natural subsequent step is to devise new research that quantifies the impact of introducing DCT elements to various systems by considering the value generated for different stakeholders. To that end, we encourage and invite opportunities to collaborate with industry stakeholders to investigate a range of topics, ranging from mapping the types of operational models related to drug distribution and management, developing a scoring tool to systematically apply DCT elements and solutions to clinical trials for various conditions, to classifying the different types of devices used and examining their impact on patient experience.

## Data Availability

Not applicable.

## Appendix

### A Discussion of the Advantages, Disadvantages, Obstacles, and Enablers Associated with DCTs

Perhaps the most significant advantage of DCTs is that they facilitate more accessible and convenient clinical trial participation. For study volunteers, this means less disruption to their daily lives, a convenient and flexible participation experience, and increased representation in clinical research. For sponsors, this results in better recruitment and retention and a larger and more diverse patient pool that offers the potential to complete trials faster and with increased external validity. For sites, DCTs can reduce staff workload and execution burden by allowing certain traditional site activities to be conducted remotely. Moreover, both sites and sponsors can benefit from the insights from real-time data collected in real-world settings. The efficiencies gained from DCTs carry the potential of significant cost advantages over time.

DCTs also pose certain disadvantages and must be implemented cautiously. Skepticism in the industry stemming from the capabilities and infrastructure that need to be built alongside the introduction of DCT solutions and the time and resources necessary before benefits are realized. For participants, DCTs require a more active role in the trial and data collection, which can be particularly challenging if participants’ digital literacy falls short of what is needed, leading to potential inequalities. For sponsors, DCT implementation may necessitate new technology, new vendors (such as home HCPs), and new operational requirements. Sites and sponsors must make great efforts to maintain patient safety and to carefully consider how to ensure data protection, oversight, and data integrity.

Perhaps supply chain challenges most threaten DCT adoption given the changes required to facilitate drug logistics and management across multiple locations, including patients’ homes. This necessitates a high degree of coordination across many stakeholders operating in different supply chain areas and various geographies. Additionally, the number of nascent technology solutions and vendors has raised concerns regarding vendor selection and reliability, ease of integration, and interoperability of systems. Moreover, due to data in DCTs coming in from a wide range of sources, the complexity of data transfer, compilation, interpretation, analysis, and management has intensified. Such difficulties threaten the promise of DCTs and diminish their associated advantages. Considering and developing means to manage complexities is critical in overcoming obstacles to DCT uptake.

Despite the various challenges, many enablers continue to spur demand for DCT solutions. Although there have been calls for clearer guidelines, more recent articles indicate growing regulatory agency commitment for DCT use in clinical trials. Indeed, the COVID-19 pandemic has resulted in increased and quickly evolving regulatory acceptance of decentralized interventions. Furthermore, the pandemic has helped improve attitudes toward digital health solutions and has heightened stakeholder comfort levels with digital technologies. These shifts in attitudes, alongside digital advances, growing sponsor and CRO investments to developing and bolstering IT infrastructure, and efforts to simplify protocol designs, will undoubtedly reinforce the continued adoption of DCTs.

#### Advantages

##### Better Recruitment and Retention

Currently, 85% of traditional clinical trials fail to recruit enough patients, and 80% are delayed due to recruitment problems [62]. Challenges with recruitment and retention can be incredibly costly for the sponsor, with direct and indirect costs reaching as high as $8 million per day [6]. Conventional trials may cause significant disruption to participants' everyday lives. In a recent study of more than 12,000 respondents patient travel was identified as the top burden to participation, “with 3 of 10 (29%) indicating that it was “somewhat” or “very burdensome” [9]. DCTs enable a greater recruitment and retention rate by allowing trial activities to occur outside sites [14]. They have shown promise, particularly during the COVID-19 pandemic. For example, a recent study found that DCT supported clinical trials were the only ones that recovered and exceeded pre-COVID recruitment rates compared to traditional clinical trials, which never fully recovered [63].

##### Enhanced Access

In conventional trials, sites tend to be located in urban areas [64]. The lack of availability of local trials serves as a barrier to participation for patients living in rural areas [2, 65]. DCTs allow more patients to have access to innovative medicines [14]. Moreover, with a larger patient pool, sponsors can benefit from a faster recruitment rate [58].

##### Increased Diversity

Relatedly, another area that can most certainly benefit from a DCT approach and the increased accessibility that it enables is patient diversity. As different patient subgroups may respond differently to therapies, the lack of diversity in clinical trials may mean that findings from a largely homogenous participant pool may not be generalizable [66]. Despite the need to include patients representing the general population, less than 5% of eligible patients participate in clinical research, and the figure is even smaller for racial and ethnic minorities, who are continually underrepresented in clinical research [10]. As previously discussed, the traditional site-centric archetype severely limits the participation of such individuals unable to travel for study visits. Such obstacles inadvertently lead to the exclusion of patient populations from underserved geographic areas. Though DCTs cannot solve all the barriers mentioned, they can mitigate some of the major challenges related to accessibility, the most obvious being DCTs’ ability to make certain clinical research studies more geographically accessible compared to traditional studies. By increasing accessibility and removing some of the financial toxicities of clinical trial participation [7], DCTs could lead to better representation and increase the external validity of trial findings [67].

##### Improved Patient Engagement

DCTs can also positively influence patient engagement—the effort and movement to amplify and address patient voices in drug development and delivery. The flexibility afforded by DCTs can enrich the patient experience [68]. A convenient trial experience can make it easier for patients to participate and remain in the trial, thereby increasing compliance and adherence [14], which may enhance study safety [69].

##### RWD and RWE

Electronic data capture gives sites and sponsors real-time access to data, which has multiple benefits. Issues can be quickly identified and addressed [68]. For instance, sites can be alerted to safety problems between on-site visits [70]. By reviewing data more quickly, sponsors can derive insights to optimize study outcomes [71] and use data to inform clinical trial design. Data collected in real-world settings while a participant goes about their daily routines may represent the patient’s experience more than data obtained through discrete site visits [72].

##### Reduction in Site Burden

DCTs allow certain traditional site activities, such as drug administration and assessments, to be conducted remotely by participants or other HCPs, reducing site investigators’ workload [69]. As a result, site investigators become free to pursue more complicated, high-value services [73]. The efficiency and resources gained can reduce the number of sites needed to meet recruitment targets for a study [14] and expand the number of trials that can be carried out simultaneously [13].

##### Cost Advantages

With DCTs, fewer research sites may be needed and this could potentially reduce the number of institutional review boards and redundant applications. As a result, costs and site-specific inconsistencies might decrease while making it easier to implement protocol adjustments [69]. Moreover, the remote collection of digital biomarkers could facilitate a reduction in trial sample sizes [7].

#### Disadvantages

##### Industry Aversion

According to Agrawal et al. [22], across sponsors, there can exist “skepticism about the urgency of adopting [DCT] approaches, internal cost pressures, lack of an established operating model for decentralization, and an increased amount of capability that must be built across asset teams, functions, digital and technology groups, and vendor management, among others". DCTs can also be burdensome to sites, particularly in the early stages of implementation. Lamberti et al. [47] found that many organizations are experiencing barriers related to effectively using DCT technologies such as ePRO and eConsent. Moreover, the digital tools meant to simplify the process may burden the sites. For example, a recent report revealed that the average site must log into more than six platforms for a single study [74], making the process cumbersome for the end user. Both studies underscore pain points related to identifying and using the right technology and highlight the importance of making it easy for end users to effectively and seamlessly use the different technologies.

##### Potential Inequalities

DCTs may introduce or exacerbate inequalities by excluding populations who do not have access to communication devices or the internet [75]. Only 45% of people have internet access in developing countries, with just 20% in the least developed countries [76]. The percentage of those connected in rural areas is three times lower than in urban areas [76]. In the US, 20% of the population does not have access to broadband or a smartphone [10]. Moreover, participants from low-income groups might not have private spaces to discuss confidential topics with clinical investigators [77]. van Rijssel et al. [78] also highlight concerns about digital literacy as a potential participation barrier, as DCTs would require patients to have a certain level of digital literacy to work with the different technological platforms. Thus, providing high-quality support through training to increase participants’ comfort and willingness to use technology will be paramount to ensuring the successful implementation of DCTs.

##### Privacy and Data Protection

It is important to maintain patient confidentiality and protect health data according to laws and regulations (such as GDPR or HIPPA), which may vary from country to country. This is so that data emerging from DCT elements, such as wearables, is not misused (e.g., potential discrimination from insurers based on cardiac activity recorded on a smartwatch) or hacked [79].

##### Patient Acceptance

DCTs place a hefty burden on the patient. For instance, van Rijssel et al. [78] pointed out that DCTs rely heavily on patients to monitor and report relevant data compared to traditional clinical trials where this is done at sites. Moreover, participants differ in their desire for human interaction, which may cause a preference for in-person visits. The relationship with study staff can be critical, especially when a participant is enrolled in a clinical trial for the first time [80]. As respondents to a patient insight survey indicated, these relationships appear to contribute to a positive experience even when the therapeutic offered no benefit to them [81]. Since one of the main benefits of trial participation is attention from experts, virtual trials may be too impersonal [82].

##### Operational Requirements

One concern with DCTs is that home care staff and patients play a more active role in trial and data collection and may not be able to provide the same level of oversight and environmental control as a principal investigator at an approved study site [83], which may lead to faulty data and flawed conclusions [79]. This has also sparked concerns regarding accountability from competent authorities since even though a home healthcare provider (which is not typically hired by the investigator) may be seeing patients, the investigator retains ultimate responsibility for the care given [84] and the data obtained. This raises the question of whether investigators are willing to delegate responsibilities to vendor staff particularly when it relates to primary and secondary endpoints. To ensure tasks can be shifted away from on-site investigators, it is imperative to provide support and training to vendor clinical research staff. A recent study showed that the level of training received by clinical research staff to interact with patients meaningfully was generally lacking [85], illustrating an area that organizations may want to pay more attention to, particularly as it relates to DCTs. Having a well-trained staff that understands how to help patients stay connected and engaged while providing accurate biometrics will no doubt be integral to ensuring the continued implementation of DCTs. Other risks to data integrity may stem from the fact that home nurses need to work with the equipment on hand. Technological failures may result in data loss without an expert to provide immediate fixes [86]. Moreover, with nurses taking patient samples to local laboratories for processing rather than having one central laboratory, data-transfer issues and potential variance in data standards across multiple laboratories might jeopardize data consistency [71]. Although samples can still be sent to a central laboratory if suppliers and shipping material are provided to home HCP, this may introduce different challenges such as longer delivery times and higher transportation costs.

#### Obstacles

##### Increased Supply Chain Complexity

A major complexity of DCTs is drug logistics and management. Unlike conventional trials, where drugs are shipped to centrally managed centers, DCTs require shipment to multiple coordinating locations (including patients' homes) [69]. Drugs need to be delivered in good quality and at the right time (often to coincide with a visit from an HCP), which necessitates substantial coordination among the supply chain, including logistics and technology providers, HCPs, and patients [87]. Issues in coordination can jeopardize the promise of DCTs. For instance, certain trial durations increased in the decentralized arm compared to the conventional setting in one study, mainly because some patients took several days to retrieve drug shipments from their local post office [14]. One advantage of DCTs is increased access (e.g., to participants living in rural areas). However, this complicates logistics as there are varying levels of infrastructure across geographies. Adding to the complexity, in global studies, IMP and other clinical trial materials must be packaged, stored, and transported to comply with the regulations in each country the shipment passes through [87].

##### IT Infrastructure

Another complexity arises from the need for new IT infrastructure. Organizations may have concerns about the cost, time, effort, and training required to acquire and implement new technology. Clinical researchers often have busy schedules and limited time to learn new software [88]. Vendor management also creates difficulties. For instance, there is an abundance of vendors. As stated by one industry professional, there are “15 different possible vendors for every single activity or step that goes into running a clinical trial”, creating a “tsunami effect” and complicating vendor selection [89]. Choosing a vendor with less experience with clinical trials can create challenges for data reliability [71]. Since data is amalgamated centrally from multiple healthcare providers using multiple health record systems, there is also a concern regarding the interoperability and ease of integration of IT systems [69].

##### Data Management

There are also complexities around data management stemming from the range and heterogeneity of data sources in DCTs [71]. The use of multiple parties in various sites with possibly different interfaces increases the security risk of a systems breach by external actors [68]. Establishing cybersecurity capabilities for executing DCTs [7] and ownership, accountability, and oversight of data are critical to ensuring data security [72]. Lastly, the range and volume of data can complicate the utilization and data management for staff. DCTs can be more complicated and time-consuming than conventional trials if staff must spend hours sifting through data and transferring data across various systems [88].

##### Staff Shortages

The world’s population has been growing and aging and there has been a rising burden of chronic disease [90]. At the same time, the healthcare workforce is also aging with a large portion set to retire. The WHO estimate a projected global shortfall of 10 million health workers by 2030 [91]. The COVID-19 pandemic exacerbated staffing problems among healthcare professionals. For instance, due to staff absences jeopardizing their ability to keep services running, some NHS trusts in England declared “critical incidents” [92]. Staffing issues also impacted clinical research. Staff constraints resulted in challenges with site initiation, monitoring activity, patient recruitment and patient care [93]. There are also lingering consequences from the pandemic. With staff working remotely or in a hybrid fashion, there has been a loss of side-by-side learning with experienced staff and a limitation in cross-coverage, both of which have negatively impacted staff recruitment and onboarding [94]. Moreover, COVID-19 caused attrition in clinical personnel. Attrition issues are attributed to “burn out… increasing clinical trial complexity, morale, lack of support (due to staff shortages) …lack of experience of new hires”, among others [95]. Furthermore, with the rapid rollout of decentralized trials, there have been reports of stress and anxiety related to the digital delivery of trials among research nurses [94]. Exacerbating the problem is the fact that training has not been widespread nor tailored creating gaps in the organizational support offered to nurses conducting remote or hybrid trails [94]. With historically high clinical trial activity and increased utilization of decentralized trial models [96], the need for adequate staffing of experienced research professionals such as clinical research nurses is paramount [97]. Decentralization (especially for disease areas such as cancer) increases demand for research nurses that have strong participant management skills to support trial designs which incorporate digital health (e.g., safety monitoring and remote data capture through wearables) [97]. However, with rising numbers of nurses leaving the workforce or retiring, and long lead times to train new personnel, some have raised concerns whether staff can facilitate the development of novel therapies through decentralized models [97].

#### Enablers

##### Regulatory Guidance and Changes

To minimize disruption to ongoing trials and clinical research during the COVID-19 pandemic, regulators such as the FDA and EMA issued guidance permitting the integration of alternative trial elements. Methods included virtual visits, remote monitoring, and self-administration of doses [98, 99]. With increased regulatory acceptance, further guidance across different countries will likely evolve [22]. Notable developments for the DCT industry include the FDA's launch of the Digital Health Center of Excellence, which “marks the beginning of a comprehensive approach to digital health technology, setting the stage for advancing and realizing the potential of digital health” [100] as well as the formation of the Decentralized Trials and Research Alliance that brings together sponsors, CROs, patient advocacy groups alongside the FDA, to promote the widespread adoption of decentralized research methods [101].

##### Protocol Simplification

Studies have found that protocol design complexity has grown rapidly [102], having detrimental implications for investigative site burden, patient burden, and clinical trial performance (e.g., longer cycle times and higher costs) [103]. Excessive data collection associated with complex protocol designs can compromise the data analysis process, increase error rates, and negatively impact data quality [104]. The shutdowns brought about by the COVID-19 pandemic provided a catalyst for streamlining study procedures to focus on what was absolutely necessary [89]. Concurrently, traditional site-based designs were retrofitted to allow for decentralization. As DCT adoption continues to grow, studies must be optimally designed upfront for decentralization [73].

##### Digital Advances and Funding Interest

There is considerable investor interest in digital health, with venture capital funding for digital health technologies exceeding investments made on all medical devices combined [67]. Technology companies, including Big Tech firms like Apple and Amazon, have moved into the healthcare market. According to Grand View Research, increasing smartphone penetration, improving internet connectivity, and advancing healthcare IT infrastructure, among other factors, are driving growth in the global digital health market (valued at USD 175.6 billion in 2021 and projected to grow at a compound annual growth rate of 27.7% by 2030 [105]). What is more, technologies for remote data collection are maturing and increasingly being validated, with more digital endpoints used as primary endpoints [22].

##### Comfort with Digital Health

Following the COVID-19 pandemic, attitudes towards digital health have improved both on the consumer and provider side. According to research by McKinsey, telehealth utilization in 2021 is 38X higher than before the pandemic. The analysis also shows that 58% of physicians continue to view telehealth more favorably now than they did before COVID-19, and 40% of consumers believe they will continue to use telehealth compared to just 11% using telehealth prior to the pandemic [106]. Comfort with digital technologies has also grown. A 2020 survey of healthcare consumers by Deloitte indicates that 42% of U.S. consumers used tools to measure and track their fitness and health (jumping from 17% in 2013). Among those using a fitness device, half shared data obtained from the technology with their doctor [107].

## Footnotes

1 Pain points are recurring problems that inconvenience stakeholders. They emerge when system demands and pressures conflict with the stakeholder’s attributes and constraints.

2 Since the coining of the term by Barry Richard in 1987, “systems thinking” has taken on various definitions (see [34] for a review of the literature). Many different systems thinking tools have emerged (e.g., root cause analysis, behavior over time graphs, and system dynamics, to name a few; see [35] for a discussion of tools). Systems thinking has been applied in numerous domains, including healthcare.

3 The following discussion offers a broad and illustrative utilization of the framework. We note that different “systems” as defined by the scope (e.g., a small-scale, phase 1 cancer study) will have unique specificities that need to be considered. Future research can begin to characterize the framework’s application to various systems.

4 Table 3 provides a non-comprehensive list of possible pain points. We also refer the reader to Apostolaros et al. [39], who outline the key stakeholder challenges to implementing DCTs as indicated in group interviews and an expert meeting of more than 50 diverse industry representatives.

5 One such example relates to the significant supply chain changes required to facilitate drug logistics and management to multiple coordinating locations, including patients’ homes. This “last-mile logistics” brings upon new challenges and necessitates a high degree of coordination across many stakeholders operating in different supply chain areas and various geographies. The issue of complexity also materializes in IT infrastructure and vendor management. The abundance of emerging technological solutions and vendors has raised concerns regarding vendor selection, ease of integration, and interoperability of systems. Moreover, due to data in DCTs coming in from a wide range of sources, the complexity of data transfer, compilation, interpretation, analysis, and management has intensified. Such difficulties threaten the promise of DCTs and diminish the associated advantages.

6 Complete decentralization may not currently be feasible. However, this could change as technologies mature and become more widely accessible. For instance, new biomarkers have emerged and are increasingly used in oncology. One such example is circulating tumor DNA (ctDNA). A major advantage of ctDNA analysis is that samples are extracted non-invasively through blood collection. This could reduce the need for on-site visits for tissue biopsies.

7 The adoption of DCT into clinical trials can help reduce some of the major barriers related to participation among underrepresented and marginalized groups, such as racial minorities. For instance, there is a common perception that a major reason why Black participants do not participate in clinical research is due to mistrust in medical science related to past ethical violation. Yet, recently, researchers have shown that mistrust related to past ethical violation was not a major reason for the lack of Black patients in clinical studies [48]. Lack of accessibility, limited access to specialty care, and time have been identified as barriers for participation among racial minorities [48]. By reducing geographic barriers, DCTs can help increase opportunities for racial minority patients to participate in clinical research. However, this needs to be coupled with health system transformations which promote a racially/ethnically diverse healthcare workforce, diversity, equity, and inclusion training for clinicians, and initiatives that foster community engagement and partnerships to engage diverse populations [49]. It is important to note that the benefits of DCTs may be limited for marginalized groups who, for example, do not have access to smart phones, internet, and/or lack a certain degree of digital literacy.
